# Naturalizing perceptual experience: the explanatory power of probabilistic computation and the holographic solution

**DOI:** 10.3389/fnhum.2026.1816314

**Published:** 2026-06-29

**Authors:** Asger Kirkeby-Hinrup, Elizabeth Ann Stoll

**Affiliations:** 1 Department of Philosophy, Lund University, Lund, Sweden; 2 Western Institute of Advanced Study, Denver, CO, United States

**Keywords:** consciousness, hard problem of consciousness, holography solution, perceptual experience, thermodynamic computation, probabilistic computation, neural computation, Thermodynamic Computation Theory (TCT)

## Abstract

When it comes to consciousness, a central problem is a lack of clarity regarding the ontological status of the phenomenon to be explained. What kind of ‘thing in the world’ is consciousness? In other words, what will a naturalization of consciousness look like? Naturalization requires that every agreed property of consciousness is made continuous with the properties admitted by the natural sciences. In short, the hard problem will remain hard until we fully understand cortical computation, and the mechanisms by which this biophysical process generates phenomenal content. Here, we evaluate the naturalization project in light of extant debates in consciousness studies. Following this, we introduce a neuro-physically grounded framework for probabilistic neural computation and show how it offers a concrete account of the naturalization of consciousness: the holography solution. The explanatory power of this approach is provided, and we identify several testable predictions. Given the scarcity of (even proposals of) naturalization of consciousness in the field, the holography solution constitutes a significant progress for at least two reasons. Firstly, it is a plausible hypothesis about the way the brain produces perceptual experience. Secondly, even if one is not convinced by our proposal, we nevertheless show that naturalization is *in fact* possible, and challenge other theories to deliver alternative accounts of what kind of ‘thing in the world’ consciousness is.

## 1 Introduction

A number of theories have been proposed to explain consciousness (e.g., Baars, 1997; Block, 2005; Cleeremans et al., 2020; Graziano and Webb, 2015; Rosenthal, 2012; Tononi et al., 2016). A significant part of contemporary debates within interdisciplinary consciousness studies (ICS) is dedicated to practices which aim to *(a)* bolster theoretical frameworks with empirical support (Block, 2007; Brown et al., 2019; Kirkeby-Hinrup and Fazekas, 2021; Lau and Brown, 2019; Lau and Rosenthal, 2011; Yaron et al., 2022), *(b)* pit theories against each other using one or more parameters (Doerig et al., 2020; Kirkeby-Hinrup and Overgaard, 2023), or *(c)* evaluate methodologies to decide between theories (Del Pin et al., 2021; Kirkeby-Hinrup and Fazekas, 2021; Melloni et al., 2021; Negro, 2024; Yaron et al., 2022). Recently, some have highlighted systemic problems with these practices and started to express doubts about whether they are even the right approach (Fink, 2016; Kirkeby-Hinrup et al., 2025a; Kirkeby-Hinrup et al., 2025b; Klein et al., 2020; Overgaard and Kirkeby-Hinrup, 2021; Sandberg et al., 2016).

We suggest that one source of the problems central to these debates is a lack of clarity regarding the ontological status of the phenomenon to be explained. One way of understanding this issue is by asking: what will a naturalization of consciousness look like? In other words, what kind of “thing in the world” is consciousness? Now, clearly, in one sense, consciousness is the most familiar phenomenon in the world. As Tononi (2008, p. 216) notes: “Everybody knows what consciousness is: it is what vanishes every night when we fall into dreamless sleep and reappears when we wake up or when we dream”. While this first-person acquaintance certainly cements that *there is* a phenomenon (something in the world), it does not do much to illuminate this ontology. Nevertheless, this consideration does highlight a critical difference between first-person and third-person access to the phenomenon which constitutes a fundamental epistemic challenge for the study of consciousness. The naturalization we offer below targets the third-person perspective. Moreover, our proposal comes with additional benefit that it also explains (in section 5) *why* subjective experience is not observable from the outside.

Contemporary debates contain significant discussion about the properties of the phenomenon, such as whether it can have non-conceptual content (Brinck, 1999; Jacobson and Putnam, 2016), whether perceptual experience is rich or sparse (Block, 2011, 2014; Knotts et al., 2019; Kouider et al., 2010), or whether consciousness contains levels or degrees (Barra et al., 2020; Bayne et al., 2016; Overgaard and Overgaard, 2010).

Comparatively, mentions of the phenomenon itself, for the most part, take as sufficient classical characterizations, overwhelmingly converging on fixing it by reference to the what-it-is-likeness of conscious experience as proposed by Nagel (1974), or with gestures towards Chalmers (1995) classical exposition of the hard problem (for just some recent examples see, e.g., Blum and Blum, 2022; Frohlich et al., 2021; Northoff and Lamme, 2020; Raccah et al., 2021; Seth and Bayne, 2022). To be clear, both Nagel and Chalmers’ work has done a lot for the field in terms of fixing the phenomenon to be explained by reference to how it subjectively appears to the individual. However, we suggest the core issue really resides with the natural sciences, and our lack of ability to describe in their terms what kind of ‘thing in the world’ consciousness is.

Ultimately, neither Nagel’s identifying instances of the phenomenon, nor Chalmers’ way of describing the problem, tell us anything about the phenomenon itself. Put differently, the concept of ‘what it is like’ can only do work in relation to determining if an individual is conscious of being in some state, or if an individual is a conscious individual. It does this by identifying a key marker of consciousness, namely that *for the individual there is something it is like*. However, this only tells us how to identify instances of consciousness, it does not tell us anything about the phenomenon itself.

One might of course advance the stronger claim: there being ‘something it is like’ is a constituent part—or otherwise necessary property—of conscious individuals or mental states. Indeed, this seems to follow analytically from the identification of consciousness with what-it-is-like-ness and the re-occurrence of the word “conscious” in the claim. Subsequently, one may suggest that identifying a necessary property or constituent part *is* in fact telling us something about the phenomenon itself. On this line of reasoning, Nagel’s view does tell us something about the phenomenon itself, namely that it is necessarily associated with what-it-is-likeness. While we are sympathetic to this line of argument and its conclusion, accepting it is inadequate to do any heavy lifting with respect to ‘naturalization’ of the phenomenon.

What we take ‘naturalization’ to entail in this context aligns roughly with the classical formulation by Petitot and colleagues where naturalization means: “integrated into an explanatory framework where every acceptable property is made continuous with the properties admitted by the natural sciences” (Petitot et al., 1999, pp. 1–2). Even if the sentence: “there being something it is like is a necessary property of conscious states” is true, this is still insufficient to tell us anything about what *kind of thing in the world* it is a necessary *property of*. This problem is essentially what underpins Chalmers’ epistemological considerations and Levine’s proposed ‘explanatory gap’ (Levine, 1983).

An example serves well to illustrate why knowing just this property is insufficient in the present context. Suppose all we had ever seen of hot air balloons were the baskets, and that somehow all the other parts of hot air balloons were invisible to us. Because our starting assumption is that everything can be naturalized, we assume that these flying baskets are part of some natural phenomenon conveniently named ‘balloonZ’. Since the flying baskets—and consequently our idea of balloonZ—are at odds with our knowledge from physics, we are very puzzled by this balloonZ phenomenon (some even call it a *hard problem*). Occasionally, we would see a basket flying across the sky and think to ourselves “that is definitely a case of balloonZ” or “all balloonZ have a basket,” but even though both these sentences are true, we are no closer to understanding the balloonZ phenomenon or even understanding how the baskets can travel across the sky. To understand the phenomenon, we need to know what kind of thing in the world the baskets are attached to (properties of) i.e., how they can be made continuous with the properties admitted by the natural sciences. Consequently, while Nagel’s and Chalmers’ work is helpful in many ways, invoking it is insufficient to address the question concerning a naturalization of consciousness in a satisfactory manner.

In the next section we evaluate the naturalization project in light of extant theories of consciousness. In section 3, we provide some background information on neurophysiology, which is needed to understand the account we will propose. In section 4 we introduce a framework for probabilistic computation and in section 5 we show how it offers a concrete account of the naturalization of consciousness: the holography solution. In section 6, we briefly highlight the explanatory power of this approach, and in section 7, we offer several testable predictions made by this new theoretical framework to highlight future directions. Finally, in section 8, we offer some concluding remarks.

## 2 Naturalization

As the field of ICS has blossomed in recent decades, a majority of researchers in the field ascribe to one of four dominant theories: Integrated Information Theory (Tononi et al., 2016), Higher-order thought theory (Brown et al., 2019), Recurrent Processing Theory (Lamme, 2020), Global Workspace theory (Mashour et al., 2020); yet the remaining minority of researchers constitute a large undergrowth of interesting theories and hypotheses, many of which are viable alternatives to the four dominant theories (Kirkeby-Hinrup and Overgaard, n.d.; Kuhn, 2024). To give just a few examples: Attention Schema Theory posits that consciousness is an internal model of how the brain is allocating attentional resources (Graziano, 2020; Graziano and Webb, 2015). SOMA, the Self-Organizing Metarepresentational Account, argues that consciousness is a theory the brain has built about itself, by observing its own processes over time (Cleeremans, 2019; Cleeremans et al., 2020). Others think that attention to certain levels of representation underpins consciousness (Prinz, 2011). Consciousness has also been conjectured to be a ‘decision to engage’ with the world and its contents (Shadlen and Kiani, 2013), or a method of updating predictive models of the world through the continuous minimization of a cost function (Friston, 2010; Friston and Kiebel, 2009; Seth and Hohwy, 2021). Purely mathematical approaches have led to the idea that consciousness is a coarse-grained holistic conceptual assembly from fine-grained processes (Blouw et al., 2016; Chang et al., 2020; Gosmann and Eliasmith, 2016; Kanai et al., 2019). Having a plethora of theories and hypotheses—*ceteris paribus*—suggests that there are yet unsettled questions in a field, and that there is significant theoretical wiggle room with respect to scope, conceptual framework—and even conception—of the explanandum (Kirkeby-Hinrup et al., 2025b).

In the empirical domain of ICS, much work in recent decades has been dedicated to the search for the so-called ‘neural correlates of consciousness’ (NCC). In line with the theoretical wiggle room, proposals for the NCC are abound in the literature with proponents of more or less every theory advancing a hypothesis about the NCC cast specifically in light of that theory (e.g., Boly et al., 2017). We will not rehash all the different NCC hypotheses here, since—despite surface similarities—the specifics are peripheral to the naturalization project. The first and most obvious reason the NCC are peripheral to naturalization is revealed by the word “correlates” in the concept of NCC. To elaborate, the search for the NCC concerns identifying neural events that merely co-vary with conscious experience, and as such tells us nothing about the phenomenon they correlate with. Returning to the balloonZ example from above, even if we determine that instances of baskets flying across the sky always co-occur (correlate) with an increased temperature of the air immediately above the basket (something we observe is not the case with non-flying baskets), this barely moves the needle on our understanding of the balloonZ phenomenon, or the mechanisms underlying it. We are no closer to understanding how the baskets travel across the sky.

In sum, correlations in and off themselves do very little explaining,^1^ a point also observed by Seth (2009) who has suggested we need instead to look for ‘explanatory correlates of consciousness’. Seth is not alone in his worries about whether the available theories of consciousness will deliver an exhaustive understanding of the phenomenon. For instance, Signorelli and colleagues posit that the majority of the field^2^ expect the brain to generate consciousness in a similar way as the liver produces bile, and conclude that to date “no viable mechanism has been identified for this process” (Signorelli et al., 2021, p. 3). More pointedly, Schurger and Graziano drive this point home when they note that we could have “a perfectly accurate description of what goes on in the brain that ‘gives rise to’ consciousness without having any clue as to why” (Schurger and Graziano, 2022, p. 2). They continue with a scathing diagnosis of the field:

“When we try to explain consciousness, we arrive at a chasm where we say ‘and then consciousness happens’ in order to magically jump over the explanatory gap. Virtually all accounts of consciousness have this in common. They walk you to the edge (from different angles) and then somehow you find yourself on the other side, without understanding how you got there” (Schurger and Graziano, 2022, p. 3).

A survey of the field (see, e.g., Sattin et al., 2021)^3^ clearly confirms the extent to which this diagnosis of Schurger and Graziano nails a central issue in ICS.

One question then is: *why* do the majority of theories of consciousness exhibit this tendency to gloss over such an essential part of the core explanandum? One possible explanation is a lack of clarity about what is on the other side of the explanatory gap. In other words, without a naturalization of consciousness, there is no solid ground on which to land on the other side of the explanatory gap, rendering it more akin to an explanatory cliff edge. From this perspective, there is a sense in which the quote above from Schurger and Graziano is not quite accurate. Rather than somehow finding yourself on ‘the other side’, a more apt description would be something along the lines of finding yourself ‘somewhere unspecified’ or finding yourself having jumped off the cliff into thin air.

Similarly, the lack of naturalization of consciousness also drives the problem in the liver-bile analogy from above. The liver and bile are naturalized concepts, in the sense that both can be fully explained in terms of properties admitted by the natural sciences, as can their relation. It is exactly because bile admits of naturalization that the example is invoked as a good example of the kind of understanding we hope to achieve for the relation between the brain and consciousness. To summarize, the hard problem will remain hard as long as we try to investigate something we have not fully defined in the terms of the properties, natural laws, and methodology we deploy to investigate it.

Schurger and Graziano (2022, p. 7) ask: “What is the minimal set of facts that we need to account for in order for our explanation to be complete?.” We submit that one entry in this set has to be what kind of (naturalized) thing in the world consciousness is. Unless there is firm ground on the other side of the gap, it cannot be crossed. In the remaining sections, we explicate the phenomenon in terms of probabilistic computation and the ‘holography solution’ which grounds perceptual experience in the biophysical properties of cortical neurons, thereby providing a comprehensive and falsifiable proposal for the naturalization of conscious experience. Given the lack of (even proposals of) naturalization of consciousness in the field, the holography solution constitutes significant progress for at least two reasons. Firstly, it is—to our minds—a plausible hypothesis about the way the brain produces perceptual experience, and if the holography solution is correct, it would clearly be extremely significant. Secondly, even if one is not convinced by our proposal, we nevertheless show that given an account of naturalization is *in fact* possible, and challenge extant theories to deliver alternative accounts of what kind of ‘thing in the world’ consciousness is.

## 3 Some background on cortical neurophysiology

Notably, perceptual experience is associated with the activation of neurons in sensory regions of the cerebral cortex (De Renzi, 2000; Garland, 2012; Griffiths et al., 1998). Meanwhile, the activation of peripheral sensory neurons or spinal reflex circuits is not sufficient to generate perceptual experience. Therefore, the mere activation of neural circuitry appears to be insufficient to achieve perceptual experience; some unique feature of cortical neuron information processing appears to be needed.

Naturalizing perceptual experience must therefore begin with understanding the biophysical properties of cortical neurons themselves. Consequently, we begin with a brief introduction to the physiological properties of neurons, then we draw attention to the unique aspects of neurons within the cerebral cortex which neuroscientists have uncovered over the past three decades.

All neurons actively pump positively-charged sodium ions out of the cell to attain a negative voltage across the neuronal membrane, called the ‘resting potential’. When upstream neurons release neurotransmitters, these molecules bind to receptors, causing an ion channel to open. The inward flow of sodium ions locally increases the membrane potential of the neuron, and when a neuron receives multiple coincident signals within a short spatiotemporal window, voltage-gated ion channels open, causing a flood of sodium ions to enter the cell (Armstrong and Hille, 1998). When the voltage threshold is reached, the neuron fires an ‘action potential’ and releases neurotransmitters into the synapse, signaling to neurons downstream (Magee, 2000).

In spinal reflex circuits, a single suprathreshold stimulus can trigger an action potential (Bialek and Rieke, 1992; Powers and Binder, 1995). In contrast, cortical neurons harness both upstream signals and random electrical noise to affect signaling outcomes (Steinmetz et al., 2000). This random electrical noise consists of stochastic ion leak across the membrane of the cortical neuron. These stochastic events constitute a critical component of the combined input signal, contributing to spontaneous membrane potential fluctuations which can push the neuron to fire an action potential (Dorval and White, 2005; Stern et al., 1997). And unlike spinal reflex circuits, which are robust to this kind of random electrical noise, cortical neurons actively manage excitation and inhibition to achieve a coordinated ‘cortical up-state’, hovering near action potential threshold and allowing random electrical noise to drive signaling outcomes (Haider et al., 2006). This computational process, which allows random electrical noise to drive signaling outcomes in cortical neurons, results in statistically random firing patterns in these cells (Insanally et al., 2019; Mendonça et al., 2016).

Neurons in a ‘cortical up-state’ fire when their receptive fields (however complex) are activated. Because the up-state is coordinated across large populations of neurons, the process results in synchronous firing. At the network level, statistically random ensembles of sparsely-distributed neurons fire together periodically (Beck et al., 2008). These network-wide events, known as gamma frequency oscillations, occur at approximately 30–100 Hertz and are correlated with cognitive function and multisensory perception. Due to these computational time constraints, stimuli presented for less than 10 milliseconds are typically not consciously perceived. There are an enormous number of studies demonstrating the relationship between gamma frequency and perceptual experience (e.g., Buzsaki and Draguhn, 2004; Csibra et al., 2000; Engel and Singer, 2001; Groen et al., 2018; Herrmann et al., 2004).

Given the statistically random outcomes at the individual neuron level and the network level, it is reasonable to model such a system in a probabilistic manner. Indeed, for the past thirty years, the field of neuroscience has employed inherently probabilistic mathematical toolkits to model statistically random cortical neuron signaling outcomes. This includes the use of Hopf bifurcations (Austin, 2008; Liang et al., 2020; Rowat, 2007), Fokker-Planck equations (Augustin et al., 2017; Ostojic and Brunel, 2011; Richardson, 2004; Schaffer et al., 2013; Vellmer and Lindner, 2021), and the use of Gaussians to model stochastic ion flux as white noise (Pyragas and Pyragas, 2024; Tatsukawa, 2025). Yet, all of these publications carefully avoid the implication that cortical neurons appear to be inherently probabilistic computational units, with the random leak or tunneling of sodium ions across the cell membrane driving a state change in the neuron itself.

In short, a sensitivity to the movement of even just a few ions when gating signaling outcomes is *the* unique biophysical signature of cortical neurons. In fact, it is impossible to effectively model cortical neuron activity without incorporating this random electrical noise (Faisal et al., 2008; Maoz et al., 2020; S. Ostojic et al., 2009; Roxin et al., 2011; Stacey et al., 2011).

Since neuronal activity in the cerebral cortex is correlated with perceptual experience, the biophysical and computational properties of these neurons may provide mechanistic insight into the necessary and sufficient criteria for a system to have perceptual experience, and these properties may act as a constraint on theories of conscious perception. In other words, a naturalized theory of conscious perception seems to require a mechanistic model of how cortical neurons probabilistically compute information.

## 4 A theoretical framework for probabilistic computation

With the above foundation in place, we can now model cortical computation with a focus on how ions interact in a probabilistic manner with the neuronal membrane to affect signaling outcomes. Rather than employing Hopf bifurcations, Fokker-Planck equations, or Gaussian white noise (as other researchers have done rather productively in the past, *op. cit.*) we employ a different approach. Here, we formally model the inherently probabilistic position and momentum of each extracellular ion as a quantum state. Accordingly, the local neuronal membrane potential is modeled as a distribution of possible voltages, dependent on the uncertain state of these ions.

In this approach, the likelihood of the neuron reaching action potential threshold can be modeled as a function of probabilistic ion movement. Consequently, we are moving away from the classical approach of modelling cortical neurons as binary computational units, in either on-state or off-state, encoding classical information. Instead, we are modeling cortical neurons as having some *probability* of switching from off-state to on-state, as a function of both upstream signals and random electrical noise. Here, each neuron is considered a two-state quantum system, encoding quantum information and dependent upon quantum computation to switch its state.

The computational and emergent properties of the system are surprisingly consistent across various perspectives, as can be shown with four distinct yet complementary mathematical toolkits used to model this quantum computational process: (1) accounting for the net amount of free energy distributed toward thermodynamic entropy during noisy cortical computation (Stoll, 2023); (2) using a Hamiltonian operator to model the quantum system (Stoll, 2024b), (3) using matrix mechanics to model ion states in relation to neural membranes (Stoll, 2022), (4) using wave mechanics to model the interference between probabilistic ion trajectories at the neural membrane (Stoll, 2026).

Starting with the first mathematical toolkit, we can account for how energy is physically distributed to entropy in a cortical neural network, during noisy coding (Stoll, 2023). Since thermodynamic entropy and computational information are both distributions of possible system states, and the interactions of ions at the neuronal membrane generate both of these quantities at once, we can model this computational process as a thermodynamic process—where all free energy, stored energy, work, and entropy are fully accounted for. In this model, cortical neurons engage in a cyclical process of information generation and compression, as free energy is distributed toward some thermodynamic quantity of information, then partially recovered as predictive value is extracted. As uncertainty is reduced and information entropy is compressed, an equivalent quantity of Gibbs free energy is released, directly affecting the Nernst membrane potential. This thermodynamic computational process results in the synchronous firing of sparsely-distributed neurons across the network at periodic intervals. And critically, this process of information generation and compression allows a cortical neural network to achieve noisy computation with near-perfect energy efficiency.

Using the second mathematical toolkit, component pure states are integrated into a physical quantity of information by populating a Hamiltonian operator (Stoll, 2024b). The Hamiltonian operator is then differentiated with respect to all perturbations to the system, yielding eigenvalues, or observables, on the boundary region of that high-dimensional probability density. In accordance with the Hellman-Feynman theorem (Esteve et al., 2010; Feynman, 1939), the resolution of the system state is paired with a spontaneous shift in charge distribution, as energy and matter are redistributed across the system. This change in ion position and charge distribution may involve crossing the neuronal membrane, thereby affecting the voltage of the neuron and the likelihood of firing an action potential.

Using the third mathematical toolkit (matrix mechanics), component pure states are represented algebraically by a density matrix. The density matrix evolves over some miniscule period of time as the system state (i.e., the neural network) is perturbed by its surrounding environment. The diagonalization of the combined density matrix environment (Stoll, 2022) yields a zero determinant, assigning eigenvalues for all components of the system at some defined point in time. This computational process of identifying linear correlations in a combined density matrix is equivalent to the extraction of predictive value from a thermodynamic quantity of information; in both cases entropy is compressed. The system state transitions from a prior probability (the past) to a posterior probability (the present) through a time-dependent computational process, in a physically-instantiated case of Bayesian inference. Notably, thermodynamic constraints imposed by the first and second law naturally limit the completeness of knowledge that can be achieved by the system (Stoll, 2024a).

Using the fourth mathematical toolkit (wave mechanics), component pure states are represented geometrically as a complex-valued wavefunction, or a high-dimensional distribution of probability amplitudes (Stoll, 2026). Here, each ion is modeled as an electromagnetic point source, or a distribution of possible positions, momenta, and atomic orbitals, rather than having some defined state. The membrane potential of each cortical neuron is then a function of these probability distributions, since the neuron encodes these ion trajectories into its own voltage state. As these wavefunctions constructively and destructively interfere, entropy is compressed, uncertainty is reduced, the system state is defined, and each ion either crosses a neuronal membrane or does not. Then immediately, a new time evolution begins and a new probabilistic system state emerges. If the probabilistic trajectory of each ion affects the voltage state of multiple computational units (i.e., multiple neurons), then the system state must be computed as a whole, with the state of every neuron being resolved as every ion state is resolved.

Each of these four mathematical toolkits describes a neural network as expending free energy to encode information, or a distribution of possible system states, then compressing that information through a physical computational process, thereby selecting an optimal system state to encode the surrounding environment. Data is gathered across multiple sensory modalities, and transmitted to cortical neural circuits, with this process of noisy coding generating quantum information. This quantity of quantum information can be described from different perspectives by energy accounting, by a Hamiltonian operator, by matrix mechanics, or by wave mechanics depending on which toolkit is employed. The computational process consists in the system identifying correlations between its own state and the state of its surrounding environment, thereby compressing this quantity of information entropy. Some uncertainty or entropy always remains, but much of this thermodynamic computational quantity can be compressed, as the system parses a signal from the noise, and encodes the state of its surrounding environment.

The amount of predictive value extracted during the computational process is equivalent to the amount of information compression *and* the amount of free energy released back into the system which can then be re-deployed to do work (Still et al., 2012; Stoll, 2022, 2023, 2024a, 2024b). This computational process obeys the first law of thermodynamics; since information entropy is a real thermodynamic quantity, it must be accounted for, so any increase in the quantity of information entropy is equivalent to the amount of free energy expended and any compression of information is equivalent to the amount of free energy returned to the system (Berut et al., 2012; Jun et al., 2014; Landauer, 1961; Yan et al., 2018). Furthermore, since the net change in Gibbs free energy is directly related to the change in Nernst membrane potential, this event directly affects neuronal signaling outcomes. This computational process allows a far-from-equilibrium thermodynamic system to find a mutually-compatible state with its surrounding environment – and encode the predicted state of its environment into its own system state – by physically computing information. And so, through this computational process, an optimal system state is ‘realized’ within the present context and is correlated with a sparse ensemble of neuronal activation.

To summarize, the system collects incoming data about its local environment, engages in noisy coding of these incoming sensory data, and then parses a signal from the noise. The neural network effectively traps energy to drive computational work, compressing entropy in both a computational sense and a thermodynamic sense. The neural network cyclically generates and compresses a physical quantity of information, reducing complex-valued probability distributions into defined outcomes, as the position and momentum of each ion affects the voltage state of each computational unit. The result is a statistically random outcome for each neuron, occurring synchronously across the network, thereby encoding a ‘prediction’ about the state of the world.

Interestingly, our proposed framework for thermodynamic computation also provides an explanation for the incredible energy efficiency of the brain. In this model, the compression of information entropy is paired with a release of thermal free energy, in accordance with the Landauer principle (Berut et al., 2012; Jun et al., 2014; Landauer, 1961; Yan et al., 2018). This released quantity of thermal free energy can then be used to do work in the system. Because this shift in Gibbs free energy is directly related to the shift in Nernst membrane potential, the local release of free energy upon information compression immediately restores the resting potential (Stoll, 2023). Essentially, when uncertainty is reduced, the system has more free energy available to do work, so it restores the resting membrane potential; when uncertainty is high, the system must expend energy to encode this uncertainty and direct further resources toward it, so the neuron fires a signal. The reduction of uncertainty is a computational process of predicting the state of the world and the capability of the body, *and the statistically random neuronal ensemble activity we observe across the cerebral cortex is encoding both that prediction and any remaining uncertainty*. As a result, this cyclical process of information generation and compression is associated with the construction of predictive models (Stoll, 2024a). This approach usefully provides a mechanistic framework for explaining the energy efficiency of the brain, and links probabilistic computation directly to the extensive neuroscientific literature on predictive processing (Hohwy et al., 2008; Kok et al., 2013; Snyder et al., 2015). We proceed in the next section, to explain how this physical process of probabilistic computation in the cerebral cortex gives rise to perceptual experience.

## 5 The holography solution

As a prelude before we delve into the details, the following overview is helpful at this stage: In this approach, perceptual experience is literally a hologram, a virtual image created by the wave-like interference of ions at the neuronal membrane. This is neither a metaphor, nor a ‘neural correlate’, but rather a biophysical mechanism for the production of phenomenal content. Simply put, the hologram is a multi-sensory percept and a natural by-product of probabilistic neural computation.

With this overview as a reference point, we now move on to the details of how holographic information content arises from the neuronal encoding process, starting with a clarification of the physical processes at work. As elaborated upon above, each ion within the extracellular space of the neurons is described as an electromagnetic point source, interacting in a probabilistic manner with each neuronal membrane. We then utilize the mechanics of holography to describe the trajectory of that electromagnetic point source (Caulfield, 2002; Colburn, 1971; Jahoda and Siemon, 1972; Lawrence and Sheridan, 2001; Ross and Shumlak, 2016). In this model, the ion is a quantum system; it has no real defined values in the present moment. Its position, momentum, and atomic orbital are intrinsically uncertain, along with the amount of time that has passed since its state was last defined. The ion is best described as a distribution of probability amplitudes across five orthogonal axes: *x, y, z, spin,* and *time*. The values on each of these axes can be represented as a distribution of probability amplitudes, since the ion does not have a defined state and cannot be pinpointed in the present moment. In other words, on this analysis the ion is a high-dimensional probability distribution; it is a wavefunction rather than a real object.

This distribution of states *is* quantum information, and the neuronal membrane potential is a function of these probabilistic ion states. This physical quantity of information is generated through noisy coding, and this quantity of information is compressed as correlations emerge between the encoding system and its surrounding environment. The constructive and destructive interference of high-dimensional probability amplitudes, or wavefunctions, results in a non-deterministic computational outcome for each neuron. If the surface of each computational unit is also an organic or synthetic charge-detecting polymer substrate which meets the established criteria for a holographic recording surface, the constructive and destructive interference of these wavefunctions will also yield a holographically reconstruction of the encoded information. In this case, this process will naturally generate a virtual ‘image’ of the information content encoded in each sensory modality.

If the probabilistic trajectory of each ion over time *t* affects the voltage state of multiple computational units, then the system macro-state must be computed as a whole, with the state of every neuron being resolved as every component pure state is resolved. The constructive and destructive interference of high-dimensional probability amplitudes results in a computational outcome for each cortical neuron, and these synchronous outcomes yield an observable system state, characterized by the periodic firing of statistically random ensembles of neurons across the network.

Here, a fine-grained encoding process gives rise to a holistic representation of the system state, due to the interdependency of probabilistic ion states across the system. Ions outside the brain are unlikely to cross the neuronal membrane, and therefore can be ignored, but each ion within the brain could cross a neuronal membrane. Each ion can be described as a wavefunction, or a distribution of high-dimensional probability amplitudes providing the position, momentum, and orbital quantum number of the ion after some amount of time has passed. The wave-like interference of these high-dimensional probability amplitudes, on the charge-detecting polymer surface of a neural membrane, yields a cohesive holographic projection of information content. This content is exclusively perceivable by the system that encodes it; the perceived content effectively *represents* incoming sensory data and *distinguishes* dissimilar sensory data. The accuracy of the perceived information content is limited by the range and sensitivity of the sensory apparatus. And critically, this perceptual content is generated as a natural by-product of cortical neuron computation. By physically encoding information, and meeting specific structural and functional criteria, cortical neural computation allows an organism to generate a holographic reconstruction of its surrounding environment, based on information gathered through its sensory apparatus.

In this model, the reference wave is the immediately previous neural network state (i.e., the position and momentum of each ion, and the resultant voltages of each neuronal membrane). The object wave is the present neural network state, i.e., the position and momentum of each ion, and the resultant voltages of each neuronal membrane. Each object wave is an ion that has undergone some change or perturbation—and again, there are many possible object waves for each ion. The interference pattern between the reference wave and all possible object waves can be considered as a function of the phase angle difference between the previous neural network states (a high-dimensional probability distribution) and the current neural network state (also a high-dimensional probability distribution). Taking the derivative of that high-dimensional volume of information yields eigenvalues, or observables.

This approach is simply a higher-dimensional extrapolation of standard holography, using ions as electromagnetic point sources rather than photons. But like standard holography, the constructive and destructive interference of the reference wave and the object wave leads naturally to a holographic reconstruction of the encoded information. A holographic projection generated by wavefunction interference is categorically different from neural activity. The neural activity *is correlated* with perceptual content, while the hologram *is* the perceptual content. Critically, the holographic projection is also categorically different from the process of encoding information and the holographic recording surface itself. This reconstructed volume of information is a virtual projection of the encoded data, and it has qualitative properties. This holographic reconstruction of sensory information is comprised of data streams from all sensory modalities – visual, auditory, tactile, olfactory, gustatory, and proprioceptive.

Notably, this holographic reconstruction is not made from light waves which are accessible to any observer, but rather quantum wavefunctions which are properties of the system itself. There is no way for an external observer of the system to replicate the reference wavefunction (i.e., the previous ion trajectories), so there is no way for an external observer to access or reproduce the actual information content encoded by the neural network. The resulting percept is subjective: This phenomenal content is defined by the range and sensitivity of each sensory apparatus and is colored by the past experience and cognitive models of the individual. When system priors are shifted, the perceived content changes, even with similar incoming sensory data. And so, as cortical neuron connectivity and ion channel properties change, with age or learned experience or pharmacological stimulation, organisms may experience shifts in perception, even when processing identical stimuli.

Importantly, in this model, perceptual experience is *not* epiphenomenal, and the generation of the hologram is mechanistically connected to the probabilistic computational outcome. The generation of the hologram is mechanistically connected with the collapse of the wavefunction as recently shown by Stoll (2026). In that work, specifically equation 19 takes the derivative of all possible values for each phase angle, with respect to all perturbing factors for each ion. Equation 20 shows this process of holographic interference to be exactly equivalent to the Hellman-Feynman equation guiding wavefunction collapse. The holographic projection or multi-sensory percept is generated as the complex-valued probability amplitudes constructively and destructively interfere, yielding a non-deterministic computational outcome for each ion (and therefore each neuronal membrane potential).

By moving away from classical, deterministic assumptions, and modeling the behavior of ions impinging on the neural membrane in a probabilistic manner, this new framework demonstrates how multi-sensory perceptual experience naturally emerges from the known physiological properties of cortical neural networks. Critically, if and only if the neural network meets the physiological criteria to undergo quantum computation—encoding information in a *probabilistic* manner, with each ion acting as an electromagnetic point source—and also meets the anatomical criteria to act as a holographic recording surface, will the system generate phenomenal content. This phenomenal content is made possible by the anatomy and physiology of the neuronal membrane.^4^ *Both* a high-dimensional holographic recording surface *and* a sensitivity to random electrical noise in gating a state change in the computational unit are critical requirements for the effect; neither feature alone is sufficient.

Firstly, the neuronal membrane must be sensitive to quantum-level noise, allowing these events to affect macro-scale outcomes at the level of the computational unit. In accordance with Max Tegmark’s original criteria for assessing whether the brain is a quantum computer, the decoherence timescales of ions must be longer than dissipation timescales of ions (Tegmark, 2000). “If τ_dyn_ < τ_dec_, we are dealing with a true quantum system, since its superpositions can persist long enough to be dynamically important. If τ_dyn_ > τ_diss_, it is hardly meaningful to view it as an independent system at all, since its internal forces are so weak that they are dwarfed by the effects of the surroundings. In the intermediate case where τ_dec_ < τ_dyn_ < τ_diss_, we have a familiar classical system.” Based on recent calculations for sodium ion decoherence and sodium ion dissipation at the neuronal membrane, cortical neurons are expected to meet the specific criteria for a quantum system, with decoherence timescales on the order of 0.4 ms, similar to the window provided by a cortical up-state (Stoll, 2022).

Secondly, the neuronal membrane—i.e. the surface of each computational unit—must also be a charge-detecting polymer surface that meets the criteria for a holographic recording surface. These physical characteristics include having: (a) a charge-detecting surface comprised of organic or synthetic polymers, with (b) a linear relationship between energy exposure and the amplitude of the reconstructed wave, to attain signal fidelity; (c) a flat spatial frequency response, to ensure signal capture; (d) a large dynamic range, to provide a good signal-to-noise ratio; (e) a high-quality and lossless material, to afford efficiency in projecting the hologram; (f) sensitivity to low energy exposure, to achieve fine signal detection; and (g) protection from environmental factors which might impact functionality. These criteria are expected to be met by the neuronal membrane (or the ion channels themselves); these charge-detecting polymer structures have high signal fidelity and signal capture, since ion movement translates effectively to shifts in neuronal membrane voltage; they have a large dynamic range, with sensitivity ranging from picoamps to milliamps; they have sensitivity to low energy exposure, allowing a small number of ions to affect a signaling outcome; they represent a high-quality and lossless material, by allowing signals to propagate with near-zero heat loss; and finally, they are protected from external factors in the environment. If these criteria are met, the process of encoding wave-like interference of high-dimensional probability amplitudes on the neural membrane should naturally yield a holographic projection of the encoded information content.

In sum, The neural membrane is a dynamic, heterogenous, asymmetric structure with nanoscale domains and rapidly changing lateral and transverse forces (Ingólfsson et al., 2017). The ion channels embedded within this structure are polymer transmembrane domain proteins exhibiting both quantum and classical molecular mechanics (Duster et al., 2016; Duster and Lin, 2019). Given these properties, it is reasonable to evaluate closely whether the neural membrane or its embedded ion channels might act as a high-dimensional holographic recording surface for encoding and projecting information.

The uniqueness of the high-dimensional holographic projection is due to several factors. Such factors include the trajectory of every ion in the system and the capacitance of each neuronal membrane, ion channel dynamics, the pharmacological effects of any circulating drugs on the neuronal voltage state, the quantity of energy available to the system for information processing, the physical location of the individual, which affects the information gathered through the sensory apparatus, and the ability for the individual to notice these sensory stimuli, given their individual experience-derived expectations, and the amount of energy they are devoting to attending a given scene or stimulus. All of these factors will contribute to the accuracy and the level of detail available in the represented information. The accuracy and completeness of the information content will also be limited by the range and sensitivity of the sensory apparatus. As such, the perception of the world, and the predictive models that are developed, will be highly subjective, and dependent upon neuroanatomical and neurophysiological features as well as life experience.

## 6 Explanatory power

In this section, we demonstrate the explanatory power found in the framework laid out above (for an overview see Table 1). The explanatory power of a theory is how it accounts for observed events, particularly empirical results that are not otherwise accounted for.

**Table 1:** Explanatory power of this new theory of consciousness.

Outstanding problem in neuroscience or philosophy	Answer within this framework	Other possible answers
The observation of surprisingly high energy efficiency in the brain, especially in the context of random electrical noise	Information entropy is cyclically generated and compressed, in a physical process of computation.	Effective distribution of energy within biological systems
The observation of statistically random signaling outcomes observed at the neuron level (i.e. interspike intervals) and the network level (i.e., neuronal ensemble activity)	The system is undergoing an intrinsically probabilistic form of computation.	Random connections lead to random outcomes.
The reported existence of qualitative perceptual content, correlated with neural activity yet categorically different from that neural activity	The information encoded by ions constructively and destructively interfering on the polymer surface of the neuronal membrane creates a holographic reconstruction.	See theories listed in Kuhn (2024) Landscape of Consciousness
Does the size of the system, or its material properties matter for consciousness?	The properties of the system are critical for achieving conscious perception. However, a system of any size with these key properties will be conscious.	Views differ.
The apparent variability of perceptual richness between individuals	Individuals have different anatomical and physiological characteristics.	The variability may be caused by individual biases, experiences and perspectives.
The apparent subjectivity of perceptual experience for individuals	The information physically encoded by the system is not accessible.	The information may in fact be decodable, if technology permits neural monitoring and processing.

Firstly, this theoretical framework resolves the outstanding mystery of the extraordinary energy efficiency of the brain, which is particularly surprising given the level of noise in the system.

Secondly, it provides a mechanistic basis for the statistically random neuronal outcomes observed at the individual neuron level (i.e., interspike intervals) and at the network level (i.e., neuronal ensembles).

Thirdly, the phenomenon of perceptual experience emerges naturally from an intrinsically probabilistic neural computation, through a mechanistic process that is solidly grounded in physics and neuroscience, under the justifiable and falsifiable assumptions that neuronal membranes contain charge-detecting polymer structures and that random noise affects cortical neuron signaling outcomes.

Uniquely, this view of consciousness is a bottom-up approach, in the sense that it does not assume from the outset that consciousness exists. It starts by formally modelling the contribution of ion ‘leak’ to cortical neuron signaling outcomes, using established toolkits in mathematical physics, and then describes the chemical properties of the neuronal membrane, in terms of meeting the criteria for a holographic recording surface. It then demonstrates how holographic projection naturally emerges from this computational process; it is not assumed to exist but rather arises from the biophysical system.

Notably, this framework implies that perceptual experience is *real*; that is to say, it is a fully naturalizable phenomenon that is physically grounded in neural events. Like bile release from the liver, this is a mechanistic process originating in biochemistry and biophysics. Returning to the example of BalloonZ, our account offers a plausible and falsifiable mechanism for baskets to float through the air. The upshot is that the physical existence of hot air balloons (a thing in the world) can be inferred from existing natural sciences, which explains the observed BalloonZ phenomenon.

Furthermore, this framework provides fine-grained processes to generate holistic content. With this approach, the richness of perception and the computational power of the system is expected to scale with the amount of energy distributed toward the production of information entropy, rather than the overall number of neurons or synapses. As a result, it should be possible for a cortical neural network that is an entire order of magnitude smaller than the normal human brain to generate perceptual experience and produce contextually-relevant behavior. We know from case studies this occurs. One example of this involves a man with hydrocephalus, who experienced fluid buildup in the brain over the course of 30 years, with no noticeable symptoms until, at the age of 44, he went to the hospital and it was discovered that 90% of his brain was missing (Feuillet et al., 2007). It appears the slow course of the injury permitted adaptation to occur, as the ventricles dilated and pressed on the cerebral cortex. Yet astoundingly, this man retained wakeful awareness, perceptual experience, and everyday function. Reasonably “any theory of consciousness has to be able to explain why a person like that, who’s missing 90% of his neurons, still exhibits normal behavior” (Axel Cleeremans quoted in Goldhill, 2016). Probabilistic computation holographic reconstruction of information as ions interfere on the polymer surface of the neuronal membrane, *can* explain this phenomenon. It also suggests that any animal with a cortex or pallium may have conscious experience. Given the conditions clarified above, a bound quantity of perceptual content will be generated by a cortical or equivalent neural network, regardless of the number of computational units or total surface area of the system.

Curiously, some people do report the absence of perceptual content. Since the theory asserts that structural properties of the neuronal membrane permit it to act as a holographic recording surface, there should be an optimal ratio of polyunsaturated fatty acids to cholesterol molecules within the lipid bilayer, and this membrane composition should determine the richness of perceptual content. Therefore, our proposal allows for a range of perceptual richness across individuals, perhaps affected by genetics or dietary factors. Correlations could be studied between self-reported perceptual richness and polymorphisms in genes controlling lipid metabolism. A mechanistic relationship could be studied by changing the ratio of cholesterol molecules to polyunsaturated fatty acid molecules within the neuronal membrane, through targeted interventions, to evaluate any link between membrane composition and reported perceptual experience.

Interestingly, this framework shows how informational content is exclusively accessed by the neural network encoding it; there is no way for an external observer to replicate the reference wavefunction (i.e., the previous ion trajectories) and therefore no way for an external observer to access the actual information content encoded by the neural network. In other words, this framework explains why perceptual content is only subjectively accessible, thereby shedding light on one of the biggest methodological problems in the study of consciousness (Overgaard, 2015; Overgaard and Kirkeby-Hinrup, 2021), but also lending credibility to the claims about subjectivity and first-person access from Nagel as discussed in the introduction above.

## 7 Testable predictions

According to our model, there are predicted quantifiable thermodynamic constraints on cortical computation. For instance, attention should be quantifiable, since the amount of attention devoted to a sensory stimulus is *literally* the amount of energy distributed toward the cortical processing of the attended stimulus. The salience of a stimulus is provided by the energetic efficiency of cortical processing. If a stimulus is particularly prominent or striking, very little net energy must be dissipated to achieve a percept.

Along with this general statement, we offer a host of directly testable predictions of our proposal, which should provide clear avenues of concrete further investigation for neuroscientists (Table 2). The testable predictions of a theory go beyond what is already observed in the field, by stating the expected results of new experiments – a systematic process which allows the theory to be falsified or to gain support through experimentation. Several predictions relate to probabilistic computation, and others relate to perceptual content as a holographic projection of encoded information.

**Table 2:** Testable predictions of this new theory of consciousness.

Proposed mechanism	Predicted observation	Current evidence	Suggested experiment
Cortical neurons compute quantum information, by physically maintaining sustained uncertainty in ion states.	Coulomb scattering profiles of sodium ions must be at least 33nm^2^ to meet this criteria. This value should be larger in cortical neurons.	Coulomb scattering profiles have been modeled in generic cells and in neurons, and these results are in line with predictions.	Cryo-electron microscopy can be used to evaluate electrostatic properties of sodium ions at the cellular membrane.
Cortical neurons compute quantum information, by physically maintaining sustained uncertainty in ion states.	Decoherence timescales of ions at the neuronal membrane should be greater than dissipation timescales of ions at the neuronal membrane. The decoherence timescales of sodium ions must be at least 0.4 ms to meet this criteria.	The calculated decoherence timescales are dependent on coulomb scattering profile, as described above, the number of sodium ions needed to trigger an action potential, and their density and velocity.	Electrophysiological methods such as patch clamping allow the measurement of voltage and current, allowing researchers to calculate how ion leak contributes to action potential generation in different neuron types.
Cortical neurons compute quantum information, by physically expending energy to generate information.	Raising the temperature of the brain (>1°C) will increase the amount of noise available, altering signaling outcomes and perceptual content.	High brain temperatures are associated with hallucinations; lower brain temperatures are associated with less awareness and content.	Other possible mechanisms, including biochemical changes to enzyme function, must be discarded in order to assert a causal link.
Cortical neurons compute quantum information, by physically recovering energy as information is compressed.	Introducing photons in the infrared spectrum (>46 𝜇m) to cortical neurons will alter signaling outcomes and perceptual content.	Biophotons have been observed in the brain during spontaneous neural activity in vivo and in hippocampal slices ex vivo.	Bioluminescence detection in vivo can be used to assess the predicted correlation with healthy activity but not ictal activity.
Structural properties of a neural membrane permit it to act as an effective holographic recording surface. Therefore changing mechanical properties of the neural membrane should affect the richness of perceptual content.	Shifting the ratio of cholesterol molecules to polyunsaturated fatty acid molecules within the membrane, or the mechanical linkage between ion channels and the lipid molecules, should affect the experienced richness of perceptual content.	No evidence to date.	Genetic evaluation of polymorphisms in ion channels and lipid enzymes – along with spectroscopic analysis of lipid membrane composition in neurons – may be statistically correlated with values on a scale of reported perceptual experience.
More deterministic signaling outcomes with increased neural activity are associated with less information.	Pharmacologically increasing glutamatergic input should reduce perceptual content while prompting motor rigidity.	Glutamatergic agonists increase neural firing, reduce perceptual content, and prompt motor rigidity.	Introducing noise in the context of glutamatergic drive should reverse the effects of the drug.
More deterministic signaling outcomes with decreased neural activity are associated with less information.	Pharmacologically increasing GABAergic input should reduce perceptual content while causing motor slackness.	GABAergic agonists decrease neural firing, reduce perceptual content, and prompt motor slackness.	Introducing noise in the context of GABAergic drive should reverse the effects of the drug.
Noisy computation generates more information and less deterministic signaling outcomes, compared with noise-robust neural circuits. More noise is associated with more perceptual content and more behavioral flexibility.	Pharmacological interventions which increase perceptual content and behavioral flexibility should be associated with more uncertainty in neuronal outcomes (e.g., more excitatory and inhibitory post-synaptic potentials prior to action potential).	Psychedelic drugs increase perceptual content and behavioral flexibility, along with changes in functional connectivity, but the underlying neural mechanisms are not well understood.	Quantifying EPSPs and IPSPs prior to each action potential, during psychedelic-induced perceptual experience, could identify a correlation between noisy coding and qualia. Introducing noise alone may test any causal link.

1) Since the theory asserts that cortical neurons sustain uncertainty to achieve quantum computation, coulomb scattering profiles of ions at the neuronal membrane should be greater than coulomb scattering profiles in other cell types. These electrostatic properties can be modeled or directly measured (Brockman et al., 2007; Vacha et al., 2009a; Vacha et al., 2009b). Formally, coulomb scattering profiles of ions at the neural membrane should have a cross-sectional area of at least 33 nm^2^ to meet this criterion.

2) Since the theory asserts that cortical neurons actually compute quantum information, decoherence timescales of ions at the neuronal membrane must be greater than dissipation timescales of ions at the neuronal membrane, for the system to achieve this computation. Both dissipation and decoherence timescales can be measured (Armstrong and Hille, 1998; Ballard and Dellago, 2012). Formally, decoherence timescales should be >0.4 ms to meet this criterion.

3) Since the theory predicts that noisy coding in cortical neural networks physically generates a thermodynamic quantity of information entropy, changes in free energy availability should affect signaling outcomes and perceptual content. While increased brain temperature during fever is commonly associated with richer perceptual content and even hallucination, there are no systematic studies on the subject. Formally, raising the temperature of the brain (>1 °C) should increase the amount of noise, altering signaling outcomes and perceptual content, yet other possible mechanisms, including changes to enzyme function or protein degradation, must be ruled out in order to assert a causal link between noisy coding and perceptual experience.

4) Since the theory predicts thermal free energy release upon quantum information compression, thermal free energy release should be observed in correlation with event-related potentials, which indicate information processing, *but not* the ictal activity associated with seizures. Biophotons have indeed been observed in the brain during normal physiological activity (Isojima et al., 1995; Kataoka et al., 2001), and introducing biophotons has been shown to drive neuronal signaling outcomes (Amaroli et al., 2018; Tang and Dai, 2014). Experimentally introducing photons in the infrared spectrum (>46 𝜇m) to a subset of neurons should alter signaling outcomes and perceptual content, in a manner dependent on the receptive field of the modulated neurons.

5) Since the theory asserts that properties of the neuronal membrane permit it to act as a holographic recording surface, changing the electrical and mechanical properties of the neuronal membrane should affect the richness of perceptual content. Neurons have been shown to have different polyunsaturated fatty acid and cholesterol content, compared with other cell types, and these properties allow for highly unstable membrane fluctuations (Ingólfsson et al., 2017). Formally, shifting the ratio of cholesterol molecules to polyunsaturated fatty acid molecules within the membrane, or the mechanical linkage between ion channels and these lipids, should affect the richness of perceptual experience.

6–8) Because the theory asserts that maintaining noisy coding is critical for perceptual experience, increasing or decreasing the likelihood of neuronal signaling outcomes should reduce the richness of perceptual content, while making neuronal signaling outcomes more uncertain should enhance the richness of perceptual content. For example, as described above, increasing the brain temperature increases noise, and should increase perceptual content, while decreasing the brain temperature decreases noise, and should decrease perceptual content. In a similar vein, pharmacologically increasing glutamatergic input should lead to more deterministic neuronal signaling outcomes, thereby reducing perceptual content and boosting motor rigidity; there is already evidence for these effects (Hanada, 2020). Meanwhile, pharmacologically increasing GABAergic input should lead to more deterministic neuronal signaling outcomes, thereby reducing perceptual content while boosting motor slackness; again there is already evidence for these effects (Campbell et al., 2014; Kapur et al., 1997; Sceniak and Maciver, 2008). By contrast, pharmacological interventions which increase perceptual content and behavioral flexibility should be associated with less deterministic neuronal outcomes, evidenced by more excitatory and inhibitory post-synaptic potentials prior to action potential). To evaluate the relationship between random electrical noise and perceptual experience, researchers might increase ion leak in the context of glutamatergic or GABAergic drive, to test whether noise is necessary to generate perceptual experience, and researchers may introduce random electrical noise in the absence of psychedelic drugs to test whether noise is sufficient to generate perceptual experience.

## 8 Concluding remarks

At the outset of this paper, we highlighted the need for naturalization in consciousness studies. Deploying the BalloonZ metaphor, we argued that common ways of fixing the phenomenon along the lines of what-it-is-likeness and the hard problem are insufficient for naturalization; a concrete biophysical mechanism is needed. Indeed, the hard problem will remain unless the phenomenon is fully defined in terms of the properties, natural laws, and methodology we expect to deploy to investigate it. Consequently, our aim is to provide an *actual* naturalization of consciousness. To this effect, we demonstrated how probabilistic computation and the holographic solution allow for an account of consciousness that is fully naturalized.

On this account, information is physically generated by a cortical neural network as each computational unit allows probabilistic events to affect the likelihood of firing a signal. A physical quantity of information is encoded into the membrane potential of each cortical neuron. The noisy encoding process naturally yields a holographic projection, i.e., perceptual content, which is representative of incoming sensory data and exclusively accessed by the encoding structure.

Individuals (cortical neural networks) collect data about the environment through their sensory systems and construct a model of the world through probabilistic coding. To this effect, the neural network expends energy to encode noisy incoming data across multiple sensory modalities, creating large quantities of von Neumann entropy. The system then identifies correlations between the system state and the state of its environment, thereby compressing this quantity of information entropy. Some uncertainty or entropy always remains, but much of this thermodynamic computational quantity can be compressed, and this energy is returned to the system as it parses signal from noise and encodes the state of its surrounding environment.

The key insight from physics that facilitated this neuroscientific theory was the finding that predictive value is both a thermodynamic quantity and a computational quantity (Still et al., 2012). The amount of predictive value extracted during the computational process is equivalent to the amount of information compression *and* the amount of free energy released back into the system to do work (Still et al., 2012; Stoll, 2023, 2024a, 2024b). This computational process obeys the first law of thermodynamics; since information entropy is a real quantity which exists in the world, any increase in the quantity of information equals the amount of free energy expended and any compression of information equals the amount of free energy returned to the system (Berut et al., 2012; Jun et al., 2014; Landauer, 1961; Yan et al., 2018). This computational process allows a far-from-equilibrium thermodynamic system to find a mutually-compatible state with its surrounding environment – and encode the predicted state of its environment into its own system state – by physically processing information.

This physical process of computation is the result of ions interacting probabilistically with each neuronal membrane, thereby affecting the voltage of the neuron and its probability of firing an action potential. The key here is to recognize that each ion is an electromagnetic point source and can be modeled as a high-dimensional wavefunction. That wavefunction is a physical form of information – a range of possible positions and momenta and energy states, distributed along the x, y, z, orbital and time axes. These wavefunctions constructively and destructively interfere on the polymer surface on the neuronal membrane, thereby encoding information. If this organic or synthetic polymer surface meets the well-established criteria for being a holographic recording surface, then this computational process should generate a holographic reconstruction of the encoded information in each sensory modality. The virtual image generated by this process would be exclusively accessible by the encoding system because no other system can recreate the reference beam, and its accuracy should be limited by the range and sensitivity of the sensory apparatus, as well as prior expectations.

This account is a neuroscientifically-grounded model of probabilistic computation in cortical neural networks: it offers a naturalized account of perceptual experience, which is a central fixture in consciousness studies. The account is non-circular, because none of the mathematical models assume that perceptual experience exists; rather they simply model electrical interactions with the neuronal membrane and follows how this noisy coding can produce holographic content through a highly energy-efficient computational process.

We appreciate that changing anyone’s mind is a tall order in consciousness studies (Kirkeby-Hinrup, Stephens, et al., 2025). However, even if one is not convinced by our account, it nevertheless constitutes a serious challenge to every other theory in the field. We have shown that consciousness *can* be naturalized, and so it is incumbent on proponents of other theories to show that their preferred theory can be made continuous with the natural sciences. Critically, this challenge asks not merely for hypotheses or data relating to supposed neural *correlates* of consciousness; the challenge concerns naturalizing the *phenomenon* itself.

In addition to this central challenge, there are several other interesting upshots of this framework which deserve highlighting here. Importantly, each of these considerations merit separate and more lengthy treatment than we are able to provide here. Consequently, each of the considerations are promising future avenues of research.

One upshot of the naturalization offered here concerns the nature of consciousness. If we are right, consciousness is a real and physically-describable property emerging from cortical neural activity. *Pace* many proposals to the opposite, the phenomenon is not underdefined or too vague to be amenable to physical laws. This framework speaks against views that consciousness is a non-existent illusory phenomenon which requires no explanation (Dennett, 1993; Kammerer, 2022; Kouider et al., 2007), and views that the hard problem is best solved by weeding out the use of mental concepts (Churchland, 1981). This framework also speaks against metaphysical positions such as Berkeley-style idealism, or panpsychism.

Another upshot follows from our focus on perceptual experience. One consequence of this focus is that the theory is currently neutral on cognitive phenomenology (Arango-Muñoz, 2019; Bayne and Montague, 2011; Montague, 2017; Pitt, 2004). Exploring possible implications in this domain would be an interesting endeavor. In a similar vein, while the physical and neuro-biological elements of the framework are well worked out, the proposed framework has similarities with other proposed theories in consciousness studies that would be worth exploring. Most prominent in this regard are the connections to predictive processing and the variational free energy principle, and engagement with main proponents of these ideas has been highly constructive (*Friston; personal communication*). In addition to this, there are similarities to other theories of consciousness that are worth exploring as well. For instance, the holography solution seems to mesh well with so-called ‘inner sense’ theories such as that proposed by Lycan (1995, 2004). Similarly, there appears to be some overlap with Benjamin Libet’s Conscious Mental Field theory (Libet, 2004). While Lycan’s and Libet’s views have not received much attention in recent years, the possible compatibility with the framework proposed here offers an incentive to reevaluate them.

Notably, our proposal stands in contrast with some other neuroscientific approaches. One example is the novel Motivated Emotional Mind (MEM) model developed by Galus and colleagues, who hypothesized that “the neuronal representations of percepts are semi-hierarchical (heterarchical), multilayered structures of the neuron-astrocyte network, in which the most frequently recurring configurations of strong neuron excitations in each layer are transmitted from the lower to the higher layer” (Galus, 2026, p. 6). Overall, MEM can be seen as an attempt to explain phenomenal consciousness through the coupling of neuronal processes with receptors, interoceptors, affect, and secondary perception. While there is room for holography projection within a MEM model, it would not serve the same role as it does in our proposal, since on MEM the explanation of phenomenal feel is intrinsically tied to sensors (Galus, 2026), whereas on the holography solution, the hologram itself *is* the phenomenal experience. Importantly, in addition to this disagreement, there are very interesting elements in MEM, such as the term “semblion” which may fruitfully be deployed to discuss neuronal avalanches of activity which are typical correlates of sensory processing (Galus, 2024, 2026).

Other theories which stand in contrast to our proposal include Global Workspace Theory and Integrated Information Theory, that each make predictions that different regions of cortex become active in different order. Critically, both GWT and IIT are still in the business of establishing *correlates* (see, e.g., Boly et al., 2017). In the absence of unique, specific, and testable predictions about the consciousness-generating process itself, it is difficult to compare these theories with our proposal. Nevertheless, we acknowledge that this literature (and more) are viable alternative neuroscientific approaches to the study of consciousness. Indeed, these approaches may productively be considered as a contrast to our approach. Clearly, our focus on the level of ion interactions with the neuronal membrane contrasts with, e.g., focuses on re-entrant sensory and interoceptive loops tied to homeostasis and allostasis. Perhaps probabilistic computation and holographic projection is not needed, and system-level neural correlates will be proven sufficient to explain the phenomenon. Critically, given that we offer testable predictions, this is a concretely empirically decidable question. Additionally, we want to stress that pending the outcomes of empirical testing, further exploring contrasts and/or intersections between our proposal and these theories certainly constitutes worthwhile future directions of research. In a similar vein, our approach may fruitfully interact with extant work on neurophenomenology, which has been instrumental in articulating questions concerning the relation between neural dynamics and, e.g., clinical outcomes with respect to non-ordinary states of consciousness (Timmermann et al., 2023).

Importantly, given that our proposal includes concrete and testable predictions it not only raises the bar for every other theory, but it is also difficult to directly compare with other theories in a like-for-like manner, if their predictions are too course-grained. For instance, we urge readers to compare the predictions offered in Table 2, with the hypothesis of higher-order thought theory, that consciousness-rendering meta-cognitive thoughts—by proxy of visibility reports in a masking paradigm—are correlated with activity in the dorsolateral pre-frontal cortex (Lau and Rosenthal, 2011).

Our proposal suggests there really was something missing from neuroscience, which made the problem of consciousness intractable for so long: information is a real physical quantity—akin to matter and energy—which must be accounted for when quantifying the total amount of energy entering the system and the total amount of work being done by the system. When a system is almost perfectly efficient – and it uses nearly all of its incoming energy to maintain its integrity, grow into a more ordered state over time, and encode the solutions to computational problems – it is not dissipating much net entropy. With this approach, we argue the system is *computing* that probability distribution, and it is recycling the energy that was used to create that probability distribution into productive work. And in doing so, it is naturally producing phenomenal content. If our brains are truly capable of undergoing this process of information generation and compression, that opens the door to a different type of computation, compared with classical computation and ultra-cold quantum computation – a type of computation where information is not merely abstract and is not controlled by a program. And if the system meets additional structural criteria, beyond noise-driven probabilistic computation, it will not only extract a signal from the noise and a meaningful prediction about the state of the world – it will also generate qualitative perceptual content which matches that prediction.

Our approach proposes an equivalency between phenomenal content and the neurally-generated holographic projection of encoded information. The rationale for this is that the holographic projection provides exclusive, subjective, informative content, and is paired with cortical neural activity. Exploring this theoretical framework may allow philosophers and neuroscientists to identify the links between neural activity and phenomenal content and build a deeper understanding of consciousness in naturalizable terms. Critically, in the present form, our proposal is neutral on the exact nature of phenomenal content. Consequently, the holography solution is amenable both to those who aims to ‘quine’ qualia (Dennett, 1992), and those who do not.

Finally, our framework allows for consciousness in non-humans. Consciousness is not a property of just any object, or even every neural network; it is associated with highly specific cortical neural network activity. Therefore, if non-human systems—either biological or engineered—exhibit the same structural and functional properties as human cortical neural networks in accordance with the above, these wetware-instantiated systems should be expected to have perceptual experience, cognitive models of the world, and the ability to output behaviors on the basis of this information.

## Data Availability

The original contributions presented in the study are included in the article/supplementary material, further inquiries can be directed to the corresponding author.

## Footnotes

1 For elaboration on this, see, e.g., the diagnoses of HOT, GWT, and IIT in Schurger and Graziano (Schurger and Graziano, 2022, pp. 2–4).

2 Specifically, they say “all materialistic or ‘physicalist’ approaches” and then continue to note that “The majority of approaches in the scientific study of consciousness endorses the position of physicalism.” (both quotes, Signorelli et al., 2021, p. 3).

3 Importantly, the survey of Sattin and colleagues is not related to the argument we are making here. However, their extensive exegesis of extant theories is perfect to underscore the point made by Schurger and Graziano.

4 The rigorous mapping onto the physics of holography is provided in Equations 1–18 of Stoll New Ideas in Psychology 2026. The connection between the Hellman-Feynman equation and the derivative of each phase angle (which directly links wavefunction interference with the compression of quantum information) is provided in Equations 19–20 in that same paper. The justification for using quantum methods is provided with references to neuroscientific papers published over the past 20 years that formally employ similar methods, such as Hopf bifurcations, Orstein-Uhlenbeck processes, Fokker-Planck equations, and the like. The question of sustained quantum coherence in the warm and wet environment of the brain remains to be evaluated in the laboratory.
